# Factors associated with seizure severity among children with epilepsy in Northern Nigeria

**DOI:** 10.4314/gmj.v56i1.4

**Published:** 2022-03

**Authors:** Idris A Adedeji, Adamu S Adamu, Faruk M Bashir

**Affiliations:** Department of Paediatrics, Abubakar Tafawa Balewa University, Bauchi, Nigeria

**Keywords:** Children, Epilepsy, Seizure Severity, Predictors, Co-morbidities, Sub-Saharan Africa

## Abstract

**Objective:**

To describe how seizure severity in children with epilepsy may be affected by certain socio-demographic and clinical variables

**Design:**

A cross-sectional study

**Setting:**

At the Abubakar Tafawa Balewa University Teaching Hospital, Bauchi, Nigeria

**Participants:**

Sixty children and adolescents who were being followed up for seizure disorder at the child neurology clinic

**Intervention:**

Information on socio-demographic characteristics was obtained with a questionnaire, details of neurological co-morbidities were extracted from the participants' records, and seizure severity was assessed with the National Hospital Seizure Severity Score 3 tool.

**Main Outcome Measure:**

Chi-square test was used to establish the relationship between categorical variables, while the Independent t-test was used in describing the differences between means. Simple linear regression was calculated to assess the predictability of seizure severity.

**Result:**

The median age was ten years (IQR = 6–13 years), with a male dominance (1.5:1). The Seizure Severity Score (SSS) ranged between 3 and 24 units, with a mean of 12.22 ± 4.29 units. The only characteristic that had a significant association with SSS on bivariate analysis was the “presence of co-morbidities” (p=0.019). A simple linear regression revealed that the presence of a neurological co-morbidity predicted an increase in the SSS by 2.67 units. [R2 = 0.091, F (1, 58)= 5.837, p = 0.019. β = 2.67, t= 2.42, p= 0.019.]

**Conclusion:**

This study shows that neurological co-morbidities predict worsening seizure severity. This knowledge may influence prognostication and the charting of a treatment trajectory.

**Funding:**

No external funding

## Introduction

Globally, epilepsy is one of the most common chronic neurological conditions in childhood. 1 Furthermore, it is known that economically disadvantaged societies harbour a relatively larger burden of this condition; this is because preventable causes such as adverse neonatal events, a higher occurrence of central nervous infections and head injury are more pervasive in these settings.^2^,^3^ In addition, the limited accessibility to antiepileptic drugs (AEDs), as well as the sparse human health resources in less developed economies, are factors that may impact negatively on the quality of care and the severity of disease of children living with epilepsy in these regions.^3^

Seizure severity measurement has long been used as an index of the effectiveness of seizure control modalities.^4^ The severity of seizures has been found to correlate with the health-related quality of life of people living with epilepsy.^5^ Describing the severity of the seizure and its correlates among children with epilepsy may enhance a better understanding of the disease and influence the management trajectory and prognostication.

The National Hospital Seizure Severity Scale (NHS3) is one of the most frequently applied tools for seizure severity assessment, which quantitatively evaluates seven seizure-related factors.^6^ These include the type of seizure, duration of time to recovery, presence of seizure-related injuries, loss of consciousness, automatism, presence of fall and incontinence. Each item is graded between 0 and 4, except for the loss of consciousness. The maximum attainable score is 27, whereas the minimum is one (1).

NHS3, which has high reliability, is quick to use and is administered by the health worker following an eyewitness interview. 6

Studies that have sought to evaluate factors associated with seizure severity and its correlates among children in our setting are very sparse. Hence, our aim was to evaluate the features associated with seizure severity among children with epilepsy in Bauchi, Nigeria. Beyond bridging a knowledge gap, our findings may herald a holistic approach toward optimising the quality of care of children living with epilepsy in this region.

## Methods

This was a cross-sectional study carried out among 60 consecutively recruited children attending the paediatric neurology clinic with an already established diagnosis of epilepsy. The study site was Abubakar Tafawa Balewa University Teaching Hospital, Bauchi, Nigeria, and the subjects were recruited over six months. Ethical approval was obtained from the Institution Research and Ethics Committee (Approval number; REC 0018/2020). Informed consent was granted by the parents of participating children, and assent was obtained from older children. Children who were accompanied to the clinic by other individuals, asides from the primary caregivers or witnesses to the seizure episodes, were excluded from the study.

Information on comorbidities of epilepsy, duration of illness and drug therapy were extracted from the patients' case files. At the same time, a semi-structured interviewer-administered questionnaire was used in obtaining details of socio-demographic parameters. The method recommended by Olusanya was utilised in classifying the socio-economic status (SES) of the parents, while the severity of the seizure was assessed using the NHS3 scale. 7

According to the International League Against Epilepsy, a seizure was defined as two or more unprovoked seizures occurring more than 24 hours apart.^8^ While for our study, an interval of 6 months or greater between the establishment of the diagnosis of epilepsy and the commencement of AEDs was considered a significant treatment delay.

Data generated were processed and analysed using IBM SPSS Statistics V 21®. The differences between the mean of continuous variables were analysed using the Independent t-test, while the chi-square test was used to establish the association between categorical variables. Simple linear regression was calculated to determine seizure severity (dependent variable) predictability by the patients' clinical characteristics (independent variable). The level of statistical significance was set at < 0.05, at a confidence interval (CI) of 95%.

## Results

The participants ranged from 11 months to 17 years, with a median age of 10 years (IQR = 6–13 years). They were mostly adolescents (55%), and there was male dominance (1.5:1). The subjects were mostly from the lower socio-economic class (56.4%) as only 10 (18.2%) and 14 (25.5%) had parents in the upper and middle socioeconomic classes, respectively. Whereas over 90% of the subjects lived with epilepsy for at least one year, only 60% had received AEDs for the same period. Indeed, the median interval between the occurrence of the first two unprovoked seizures and the commencement of AEDs was 21 months. Most of the children were on monotherapy, while only 16.7% received two or more AEDs (Table 1).

**Table 1:** Clinical and Socio-demographic Characteristics of Subjects

	Male	Fem	Total	χ^2^	P-value
Age group (years)					
<9	14	11	25 (41.7)	.283	.593
≥10	22	13	35 (58.3)		
SES					
Upper	4	6	10(18.2)	4.793	.091
Middle	6	8	14(25.5)		
Lower	22	9	31(56.4)		
Duration of illness (years)					
<1	2	2	4 (6.7)		1.000#
≥1	34	22	56 (93.3)		
Duration of AED use(years)					
<1	15	9	24 (40)	.104	.747
≥1	21	15	36 (60)		
The interval between first seizure and commencement of AED (months)					
<6	14	9	23(38.3)	.012	.914
≥6	22	15	37(61.7)		
Number of AEDs					
Single	29	21	50(83.3)	.500	.480
Multiple	7	3	10(16.7)		
Co-morbid conditions					
Present	12	10	22(36.7)	.431	.512
Absent	24	14	38(63.3)		

# Fisher's exact

**Figure 1:**
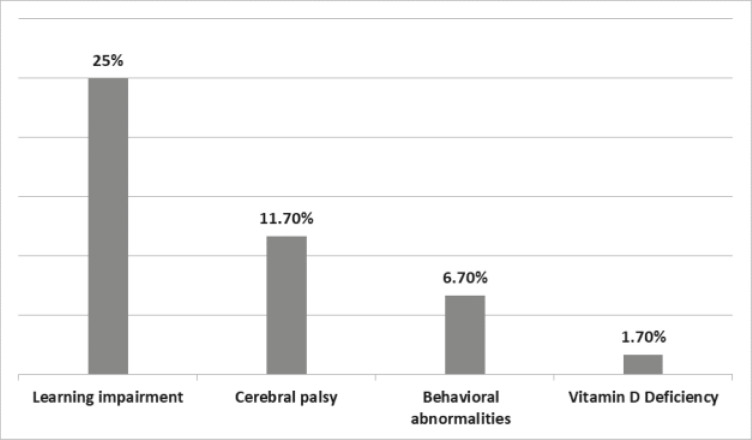
Prevalence of different co-morbidities among subjects

Co-morbid conditions were documented in 63% of the subjects; cerebral palsy, learning impairment, and behavioural disorders were the prominent co-morbidities (Figure 1).

The seizure severity scores of the subjects ranged between 3 and 24, with a mean score of 12.22 ± 4.29. Factors associated with high seizure severity score are: male gender, preadolescent age, having epilepsy for at least one year, being on AEDs for a year or more and a presence of significant treatment delay.

Similarly, children on multiple AEDs and those with documented co-morbidities also recorded higher scores. However, on bivariate analysis, the only characteristic that had a significant association with seizure severity scores was the ‘presence of co-morbidity (Table 2).

**Table 2:** Characteristics of subjects and the SSS

Variable		Mean seizure severity score	T-test	*p*- value
Gender	Male	12.72	1.119	.268
	Female	11.75		

Age group	0–9 years	13.20	1.388	.171
	≥10 years	11.57		

Duration of illness	<1 year	10.00	1.070	.289
	≥1year	12.38		

Duration of AED use	<1 year	12.20	.012	.990
	≥1year	12.22		

The interval between diagnosis and AED commencement	<6months	12.09	.183	.855
	≥6months	12.29		

Number of AED	Single	11.98	.954	.344
	Multiple	13.40		

Presence of comorbidities	Yes	13.90	-2.416	.019*
	No	11.24		

* Statistically significant

Simple linear regression was used to assess if the presence of co-morbidity significantly predicted the seizure severity score. The result of the regression suggested that the presence of comorbidity explained 9% of the variance, R^2^ = 0.091, F (1, 58)= 5.837, p = 0.019. The presence of comorbidity significantly predicted seizure severity score, β = 2.67, t= 2.42, p= 0.019.

## Discussion

This study, which evaluated seizure severity and its determinants among children and adolescents with epilepsy, revealed a mean seizure severity score of 12.22 ± 4.29. Lundgren et al., using the same tool, had reported a higher score of 15 among children with refractory epilepsy. 9 This current study comprised a mixed population of children with epilepsy, with varying degrees of response to AEDs, which probably explains the comparatively lower mean seizure severity score we recorded. This also appears to further corroborate the NHSS3 as a reliable response index to AEDs.^6^ The current study also reported marginally higher, albeit, none statistically significant, scores among males. In an earlier study by Abebayo et al., and another by Imam et al., there were no significant differences between the seizure severity scores of the two genders.^10^,^11^ This perhaps shows that despite the evidence suggesting epilepsy to be a more common condition among the male sex, its severity may have no gender bias. 12

This study found that about 62% of the children had a gap of six months or greater between the onset of the disease and the initiation of AEDs. This group of children also had a comparatively higher mean seizure severity score. Previous studies have identified a high rate of treatment gap among individuals with epilepsy in low and middle-income countries.^13^ This has been attributed to many factors, including the lack of accessibility and affordability of AEDs.^13^ It is also known that a delay in the commencement of AEDs is associated with higher seizure frequency and adverse clinical outcomes.^14^

However, we did not observe a significant difference between the scores of children who had delayed treatment and those who did not, which our limited sample size may partly explain. Also, the effect of treatment delay on seizure severity may have been subdued since most (60%) of our study subjects, including those with a history of significant treatment delay, had been on AEDs for a year or more prior to this study.

Based on the hospital records of the study participants, over a third of the children had at least one seizure-related co-morbidity, the commonest being a learning disability. Indeed, the children with co-morbidities had a significantly higher mean severity score than those without. Furthermore, this study found the presence of co-morbidity to be a predictor of seizure severity score on regression analysis. The presence of co-morbidities may indicate shared underlying neuropathology or possible development of maladaptive synaptic plasticity that leads to imbalances of excitation and inhibition, which may subsequently contribute to learning and behavioural difficulties among individuals with epilepsy.^15^ Thus, an interesting area of future research would be an attempt to establish the link between aetiopathogenesis of epilepsy, the manifestation of different neurological co-morbidities and how this relates to the severity of seizures among children.

### Limitations

It was difficult to ascertain if standardised tools were adequately applied in establishing the presence of neurological co-morbidities among the subjects, as information on these was retrieved from the case records.

## Conclusion

The only predictor of seizure severity found in this study was the co-existence of neurological co-morbid conditions. This information may help in the stratification of children with epilepsy and prognostication. Furthermore, to improve the seizure severity outcome of children with epilepsy in sub-Saharan Africa, future studies are needed to fully understand the aetiopathogenesis of seizures, especially among those that present with neurological co-morbidities.

## Figures and Tables

**Figure 2:**
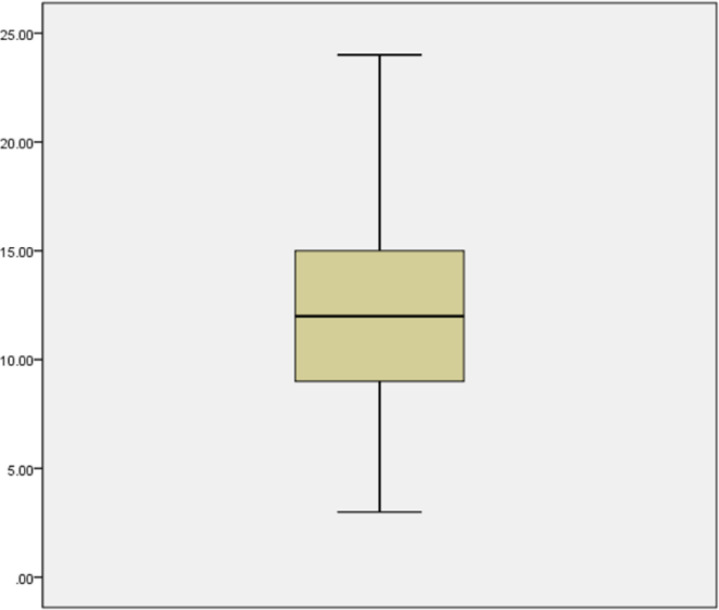
Box-plot showing the seizure severity score (SSS) of the subjects
